# Supplementary material for: Comprehensive T cell repertoire characterization of non-small cell lung cancer
Source: Nat Commun. 2020 Jan 30;11:603. doi: 10.1038/s41467-019-14273-0 (PMC6992630; doi:10.1038/s41467-019-14273-0)
Supplement: Supplementary file 1 — Supplementary Information [file 41467_2019_14273_MOESM1_ESM.pdf]

Supplementary Information

Comprehensive T cell repertoire characterization of non-small cell lung cancer

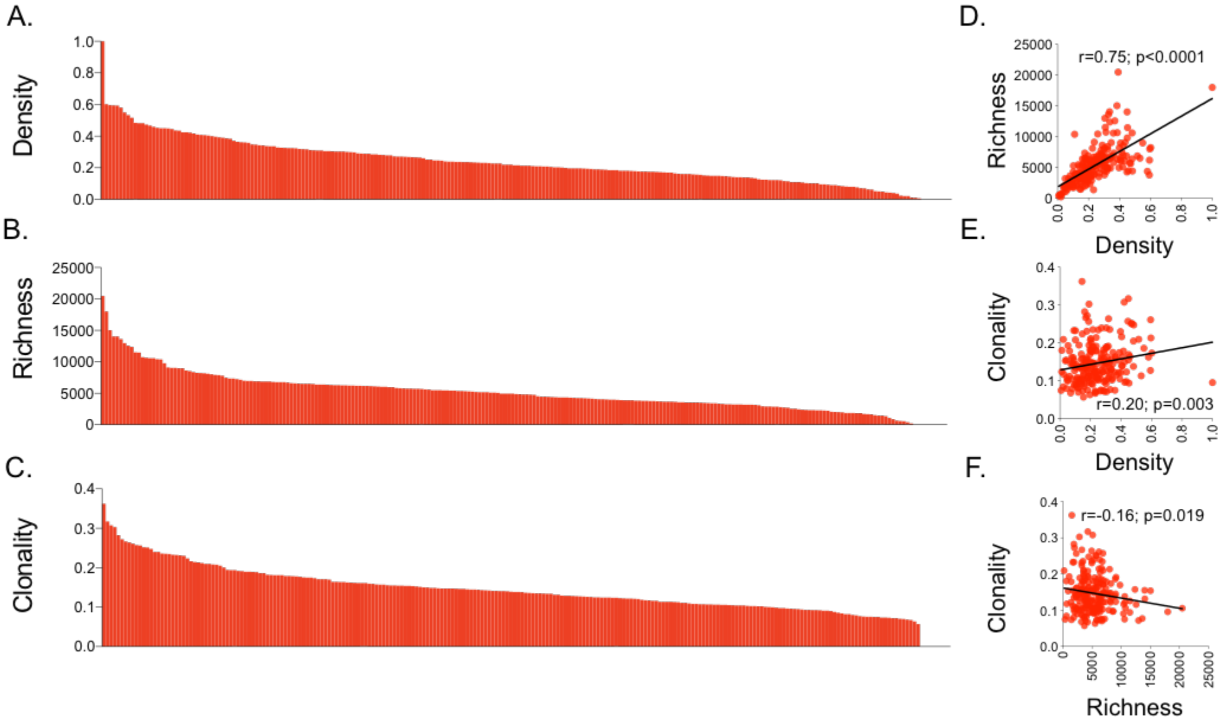

Supplementary Figure 1. T cell density, richness, and clonality are intercorrelated.

A) Estimated T cell density as measured as the proportion of T cells in each tumor sample. **B)** T cell richness as quantified by the unique TCR rearrangements in each tumor sample. **C)** T cell clonality in each tumor sample. Correlation between **D)** T cell density and richness, **E)** T cell density and clonality, and **F)** T cell richness and clonality.

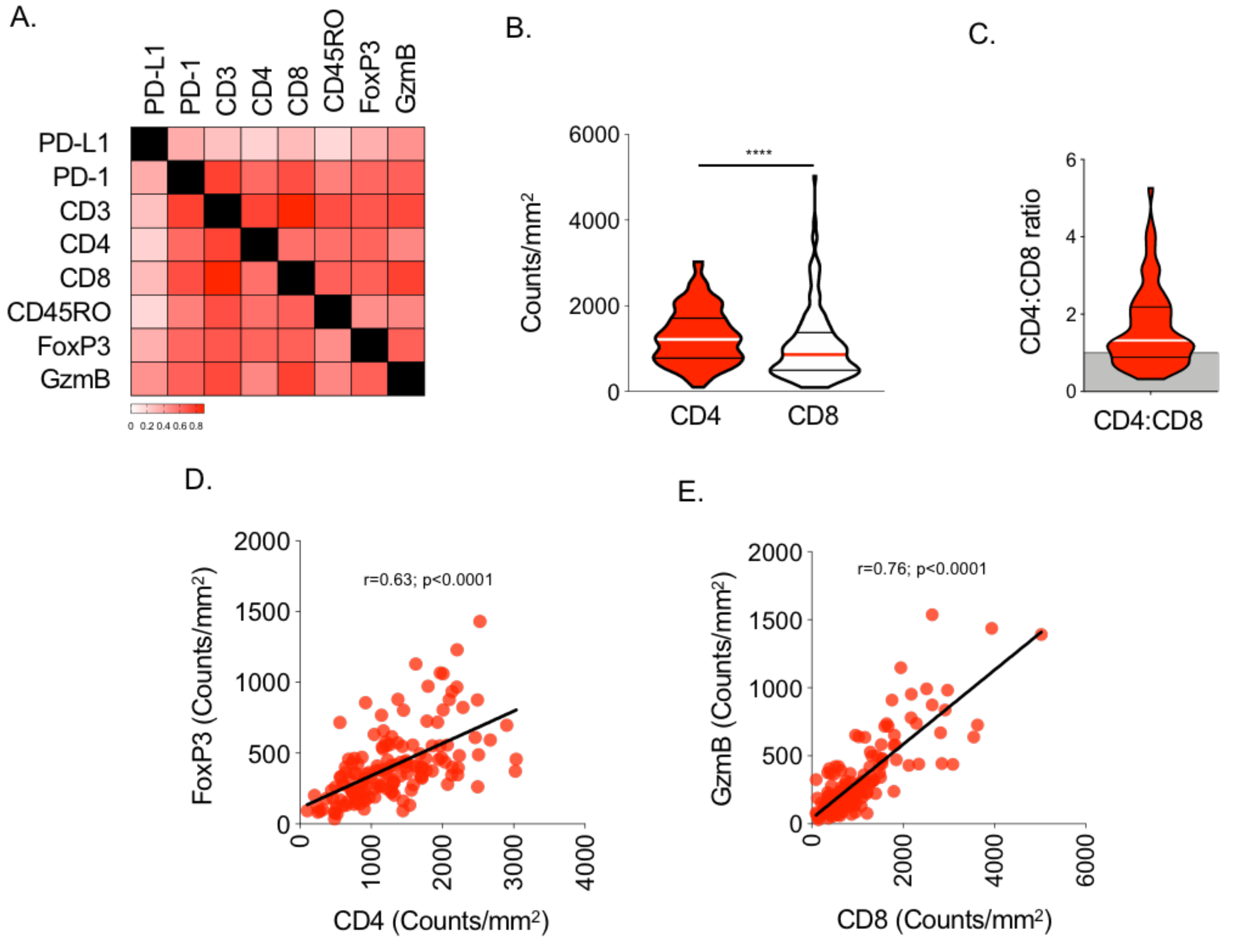

Supplementary Figure 2. Regulatory and cytotoxic T cells are major components of the tumor microenvironment in NSCLC. A) Heatmap depicting the correlation between the density of evaluated T cell markers. **B)** Number of CD4 and CD8 T cells per mm^2 in each tumor sample. **C)** Ratio of CD4:CD8 in each tumor sample. **D)** Correlation between CD4 and FoxP3 density. **E)** Correlation between CD8 and GzmB density. Bars represent median and quartiles.

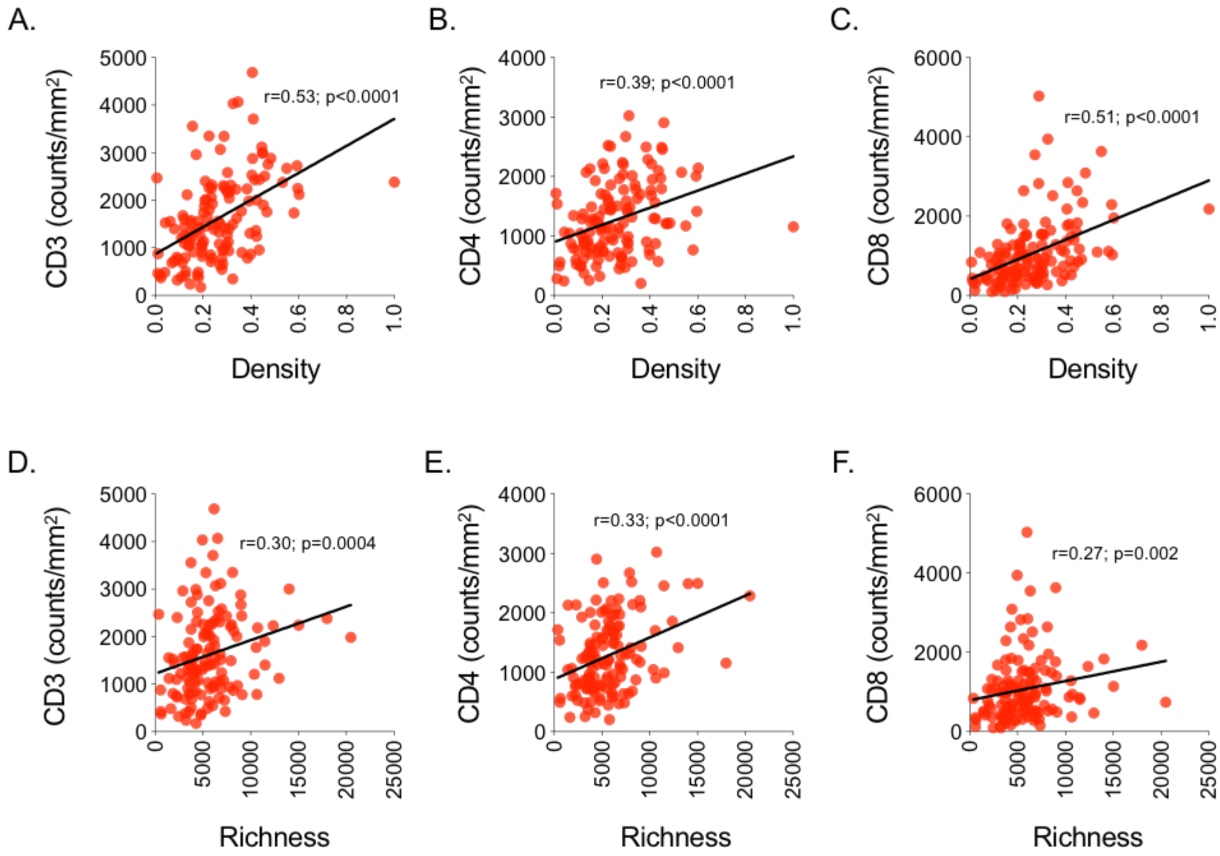

Supplementary Figure 3. T cell density and richness are correlated with both CD4 and CD8 density. Correlation between T cell density by TCR sequencing and **A)** CD3, **B)** CD4, and **C)** CD8 density by IHC. Correlation between T cell richness by TCR sequencing and **D)** CD3, **E)** CD4, and **F)** CD8 density by IHC.

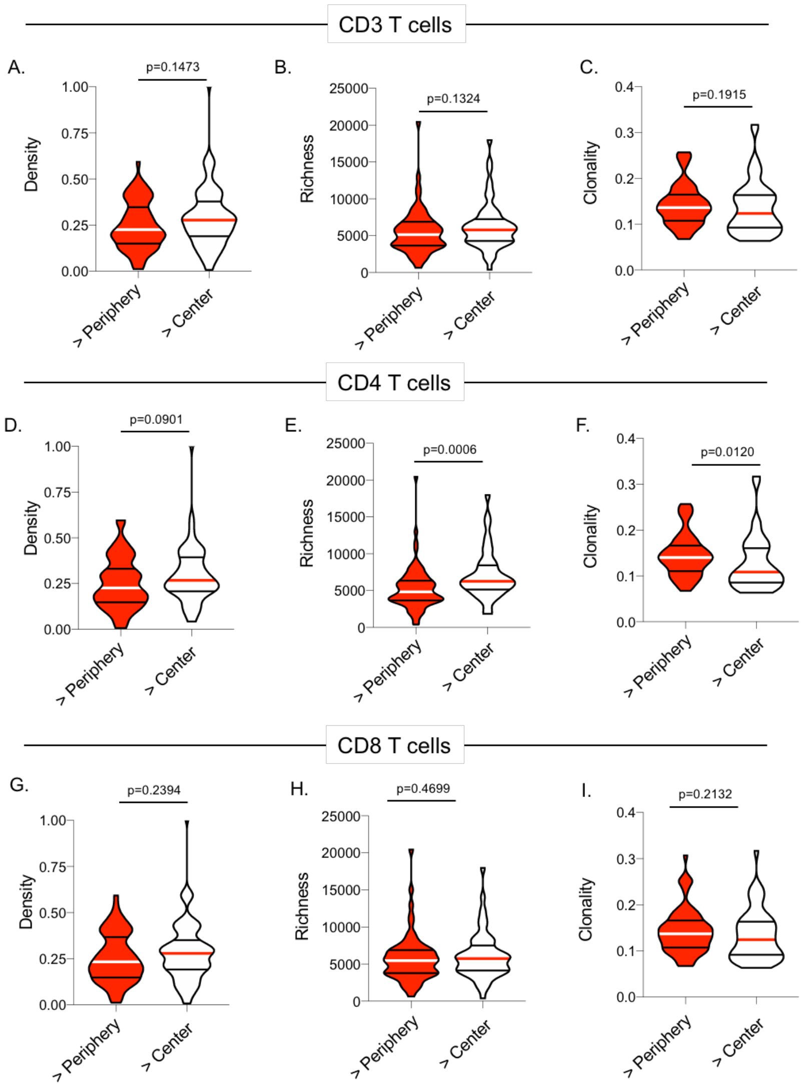

Supplementary Figure 4. T cell clonality is highest in tumors with a low CD4 density. **A)** T cell density, **B)** richness and **C)** clonality in tumors with higher CD3 density at the periphery or center of the tumor. **D)** T cell density, **E)** richness and **F)** clonality in tumors with higher CD4 density at the periphery or center of the tumor. **G)** T cell density, **H)** richness and **I)** clonality in tumors with higher CD8 density at the periphery or center of the tumor. Bars represent median and quartiles.

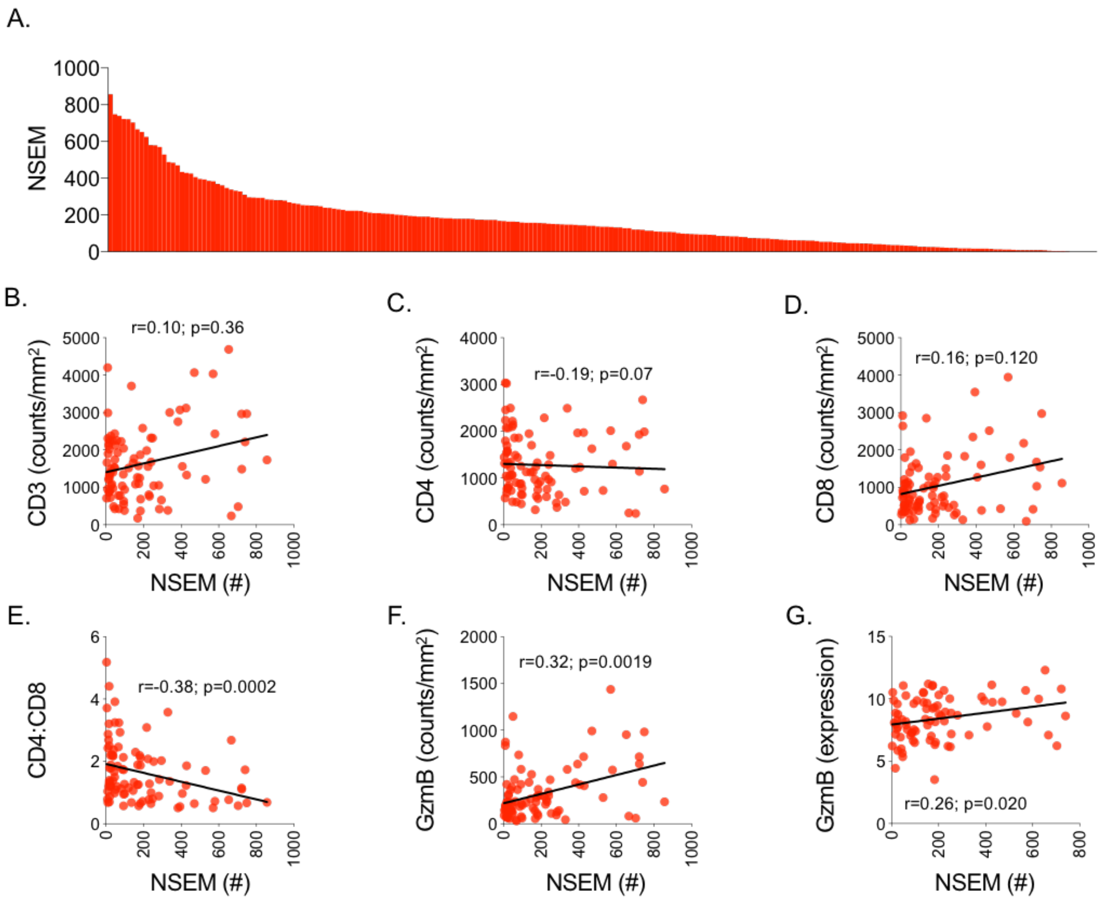

Supplementary Figure 5. An increased tumor mutational burden is associated with improved quality of the T cell response. A) Tumor mutational burden (non-synonymous exonic mutations - NSEM) in non-small cell lung cancer tumors by whole exome sequencing. Correlation between tumor mutational burden and **B)** CD3 density, **C)** CD4 density, **D)** CD8 density, **E)** CD4:CD8 ratio, **F)** GzmB density by IHC and **G)** GzmB expression by GEP.

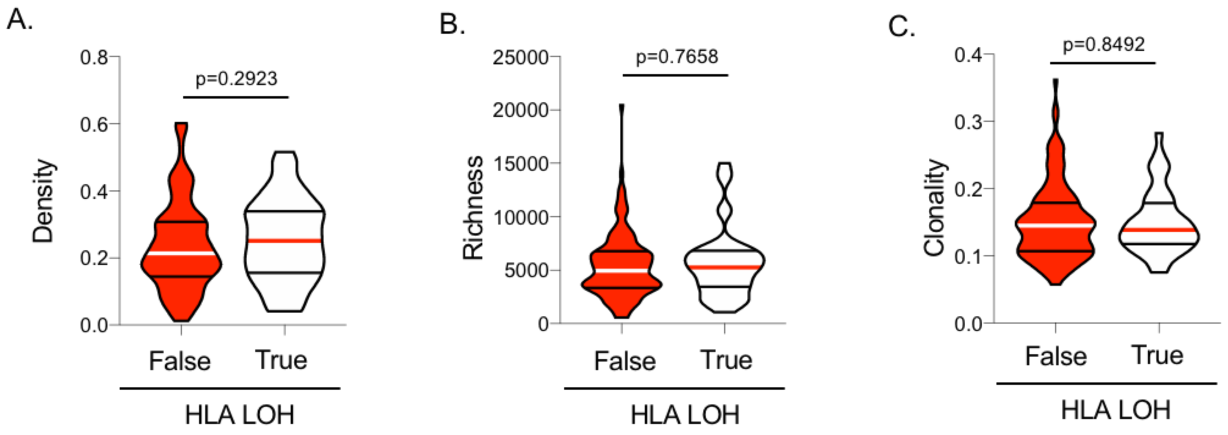

Supplementary Figure 6. T cell clonality is not associated with loss of HLA heterozygosity. A) T cell density, **B)** richness and **C)** clonality in tumors exhibiting loss (true) or no loss (false) in HLA heterozygosity (LOH). Bars represent median and quartiles.

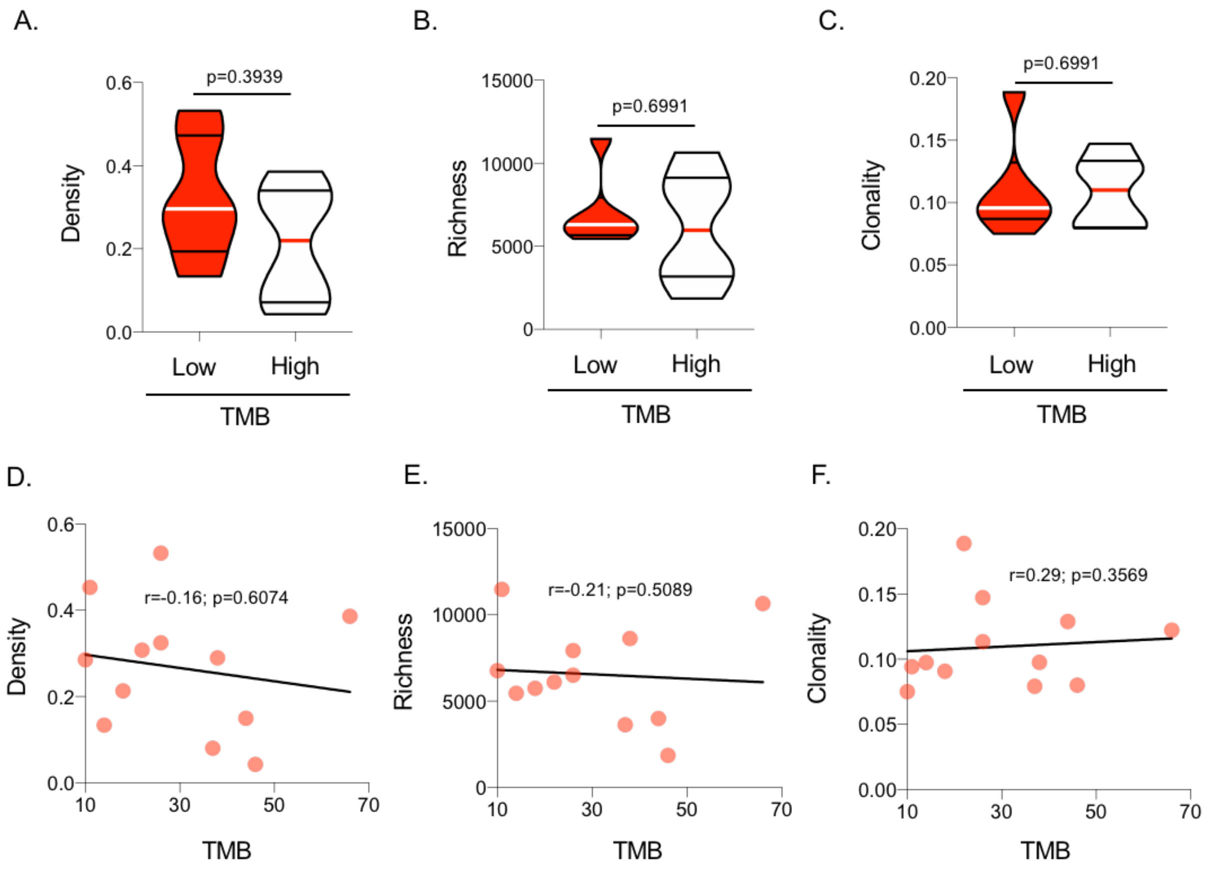

Supplementary Figure 7. The T cell repertoire is not associated with tumor mutational burden in tumors with EGFR mutations. Comparison of **A)** T cell density, **B)** richness and **C)** clonality in *EGFR* mutant tumors with a high (white) or low (red) TMB. Correlation between TMB and **D)** T cell density, **E)** richness and **F)** clonality in *EGFR* mutant tumors. Bars represent median and quartiles.

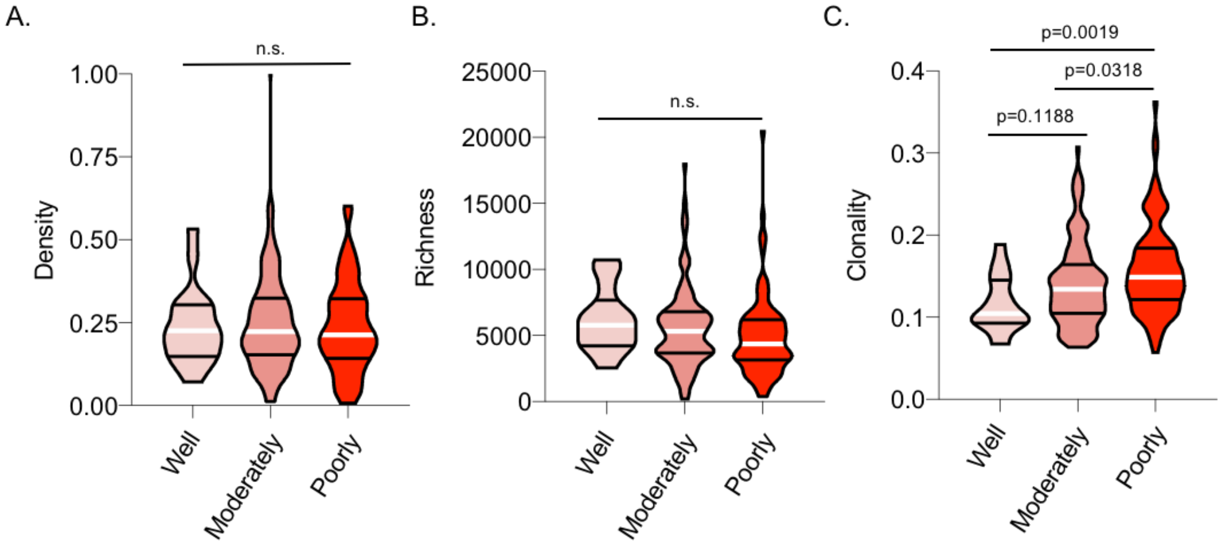

Supplementary Figure 8. Increased T cell clonality is observed in poorly-differentiated NSCLC tumors. A) T cell density, **B)** richness and **C)** clonality in well, moderately and poorly-differentiated NSCLC tumors. Bars represent median and quartiles.

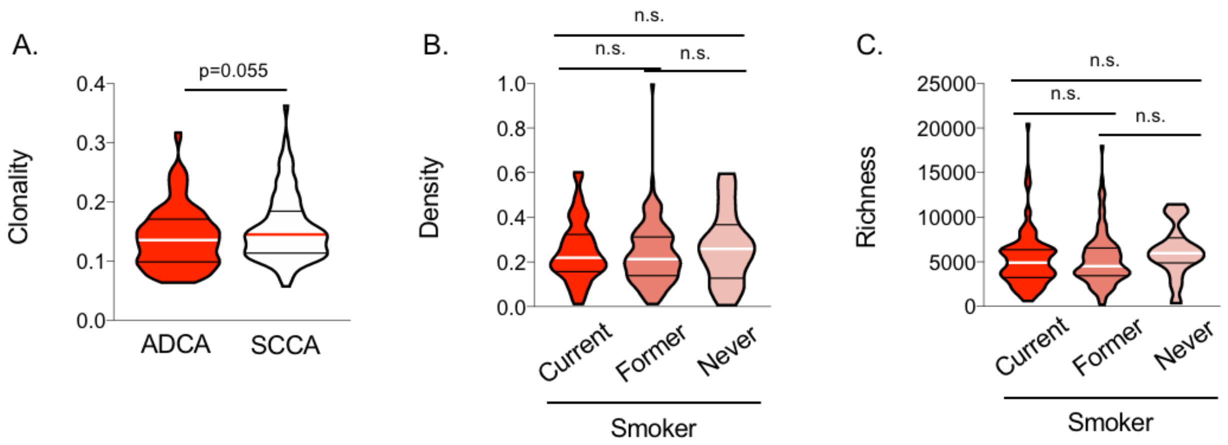

Supplementary Figure 9. The T cell repertoire is associated with clinicopathologic attributes. A) T cell clonality in patients with adenocarcinoma (ADCA) and squamous cell carcinoma (SCCA). **B)** T cell density and **C)** richness in current, former, and never smokers. Bars represent median and quartiles.

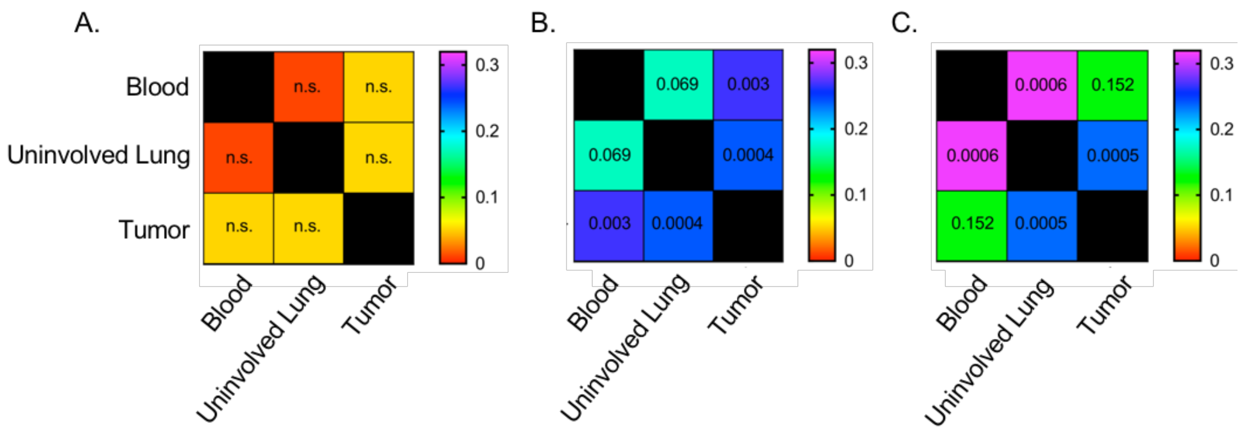

Supplementary Figure 10. Diversity and clonality of the T cell repertoire are correlated across the peripheral blood, tumor-adjacent uninvolved lung and tumor. Correlation between **A)** T cell density, **B)** T cell richness and **C)** T cell clonality across the peripheral blood, tumor-adjacent uninvolved lung, and tumor. p-values are listed in each cell.

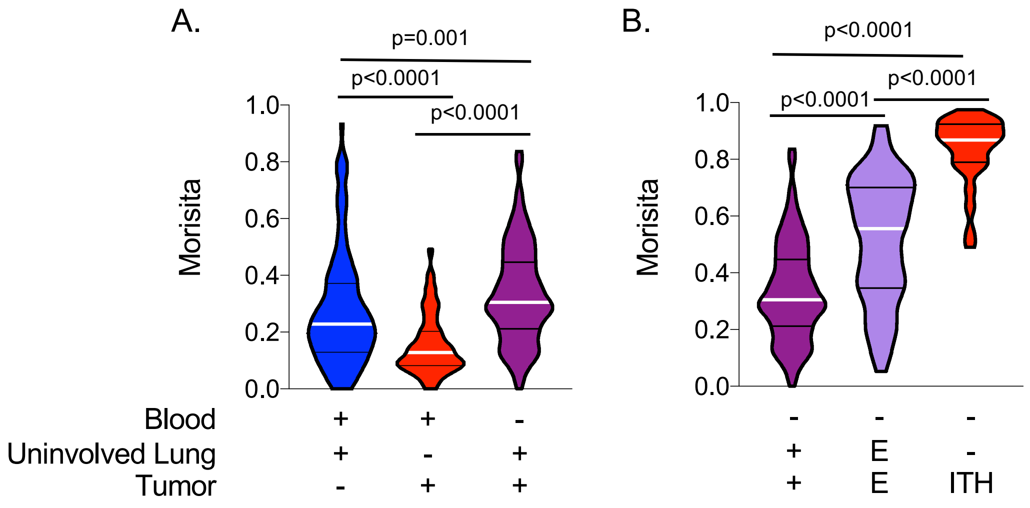

Supplementary Figure 11. Substantial T cell repertoire homology between the tumor-adjacent uninvolved lung and tumor. A) Morisita Overlap Index in the T cell repertoire between PBMC, tumor-adjacent uninvolved lung, and tumor. **B)** Morisita Overlap Index in the T cell repertoire when comparing the tumor-adjacent uninvolved lung to tumors, lung-enriched (E) T cell repertoire between the tumor-adjacent uninvolved lung and tumor, and different regions of the same tumor (ITH). Bars represent median and quartiles.

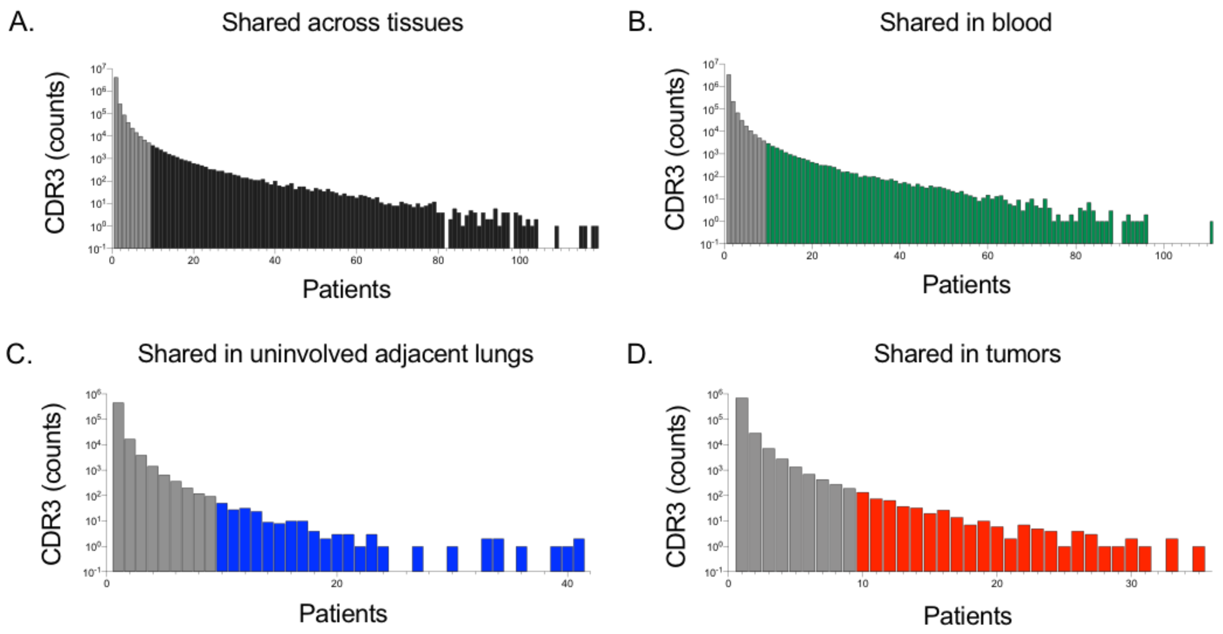

Supplementary Figure 12. A substantial number of CDR3β TCR sequences are shared across patients with NSCLC. Number of CDR3β TCR sequences shared across **A)** all tissues in all patients, **B)** only in blood samples, **C)** only in uninvolved adjacent lungs, or **D)** only in NSCLC tumors.

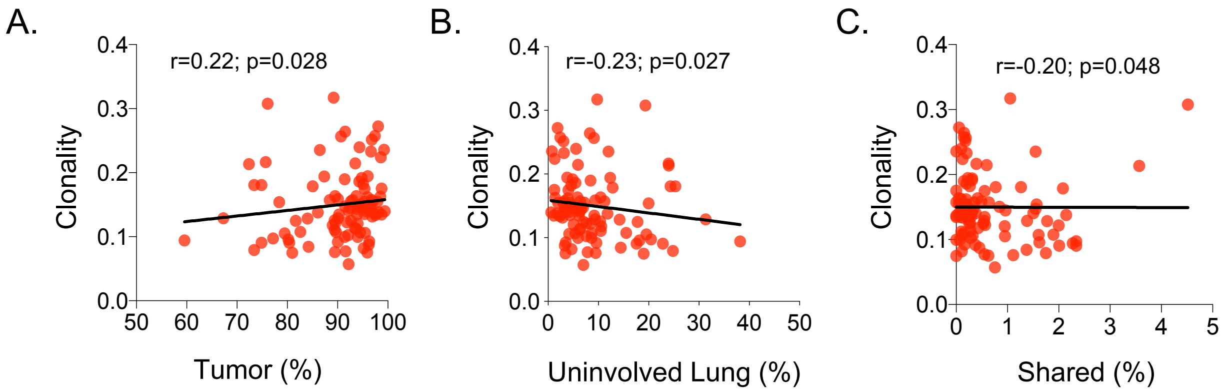

Supplementary Figure 13. Mutations in the tumor and uninvolved lung impact the reactivity of tumor-infiltrating lymphocytes. Correlation between T cell clonality in the tumor and the proportion of mutations **A)** unique to the tumor, **B)** unique to the tumor-adjacent uninvolved lung, or **C**) shared between the tumor and tumor-adjacent uninvolved lung.

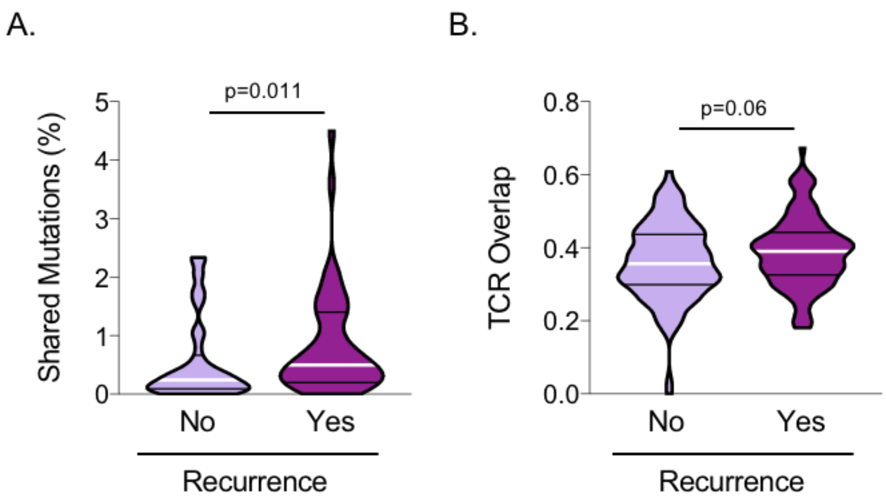

Supplementary Figure 14. A more tumor-focused lung T cell repertoire is associated with improved outcome. A) Proportion of mutations and **B)** T cells shared between the tumor-adjacent uninvolved lung and tumor in patients who relapsed and those who did not. Bars represent median and quartiles.

Supplementary Table 1. Samples and analyses included in our study.

Assay	Tissue		
	Blood	Uninvolved Lung	Tumor
Whole exome sequencing	96	222	222
RNA microarray	---	---	141
Immunohistochemistry	---	---	146
TCR sequencing	120	216	236
GLIPH	---	168	168
