# Supplementary material for: Comprehensive T cell repertoire characterization of non-small cell lung cancer
Source: Nat Commun. 2020 Jan 30;11:603. doi: 10.1038/s41467-019-14273-0 (PMC6992630; doi:10.1038/s41467-019-14273-0)
Supplement: Supplementary file 4 — Reporting Summary [file 41467_2019_14273_MOESM4_ESM.pdf]

nature research

Corresponding author(s): Jianjun Zhang, Andy Futreal, Ignacio Wistuba, Mark Davis

Reporting Summary

Nature Research wishes to improve the reproducibility of the work that we publish. This form provides structure for consistency and transparency in reporting. For further information on Nature Research policies, see Authors & Referees and the Editorial Policy Checklist.

Statistical parameters

When statistical analyses are reported, confirm that the following items are present in the relevant location (e.g. figure legend, table legend, main text, or Methods section).

n/a	Confirmed	
☐	☒	The exact sample size (n) for each experimental group/condition, given as a discrete number and unit of measurement
☐	☒	An indication of whether measurements were taken from distinct samples or whether the same sample was measured repeatedly
☐	☒	The statistical test(s) used AND whether they are one- or two-sided<br>*Only common tests should be described solely by name; describe more complex techniques in the Methods section.*
☐	☒	A description of all covariates tested
☐	☒	A description of any assumptions or corrections, such as tests of normality and adjustment for multiple comparisons
☐	☒	A full description of the statistics including central tendency (e.g. means) or other basic estimates (e.g. regression coefficient) AND variation (e.g. standard deviation) or associated estimates of uncertainty (e.g. confidence intervals)
☐	☒	For null hypothesis testing, the test statistic (e.g. F, t, r) with confidence intervals, effect sizes, degrees of freedom and P value noted<br>*Give P values as exact values whenever suitable.*
☒	☐	For Bayesian analysis, information on the choice of priors and Markov chain Monte Carlo settings
☒	☐	For hierarchical and complex designs, identification of the appropriate level for tests and full reporting of outcomes
☐	☒	Estimates of effect sizes (e.g. Cohen's d, Pearson's r), indicating how they were calculated
☐	☒	Clearly defined error bars<br>*State explicitly what error bars represent (e.g. SD, SE, CI)*

Our web collection on statistics for biologists may be useful.

Software and code

Policy information about availability of computer code

Data collection	No custom software was used to collect the data in this study.
Data analysis	The GLIPH algorithm was used to analyze TCR specificity motifs. This algorithm was published in Nature in 2017 (Glanville et al.).

For manuscripts utilizing custom algorithms or software that are central to the research but not yet described in published literature, software must be made available to editors/reviewers upon request. We strongly encourage code deposition in a community repository (e.g. GitHub). See the Nature Research guidelines for submitting code & software for further information.

Data

Policy information about availability of data

All manuscripts must include a data availability statement. This statement should provide the following information, where applicable:

- Accession codes, unique identifiers, or web links for publicly available datasets
- A list of figures that have associated raw data
- A description of any restrictions on data availability

WES data are being deposited to EGA (in progress). RNA microarray data are available in the GEO repository (GSE42127). TCR sequencing data are available on the immuneACCESS platform (DOI: 10.21417/AR2019NC). The source data underlying Figures 1-4 and Supplementary Figures 1-19 are provided as a Source Data file.

Field-specific reporting

Please select the best fit for your research. If you are not sure, read the appropriate sections before making your selection.

☒ Life sciences ☐ Behavioural & social sciences

For a reference copy of the document with all sections, see nature.com/authors/policies/ReportingSummary-flat.pdf

Life sciences

Study design

All studies must disclose on these points even when the disclosure is negative.

Sample size	Sample size was determined based on the maximal number of available samples.
Data exclusions	There were no data exclusions.
Replication	Experimental findings are based on a large patient population. Measures were not repeated, variability arises from inter-patient variability.
Randomization	No randomization was done for this study, patients were divided for comparisons based on their clinicopathologic characteristics and based on overall survival.
Blinding	No blinding was used for this study.

Materials & experimental systems

Policy information about availability of materials

n/a	Involved in the study	
☒	☐	Unique materials
☒	☐	Antibodies
☒	☐	Eukaryotic cell lines
☒	☐	Research animals
☐	☒	Human research participants

Human research participants

Policy information about studies involving human research participants

Population characteristics	Covariates of the patient population included age, gender, tumor histology, tumor size, and smoking status, and are included in Extended Data Table 1.

Method-specific reporting

n/a	Involved in the study	
☒	☐	ChIP-seq
☒	☐	Flow cytometry
☒	☐	Magnetic resonance imaging
